# Comparative Analysis of Fracture Resistance between CAD/CAM Materials for Interim Fixed Prosthesis

**DOI:** 10.3390/ma14247791

**Published:** 2021-12-16

**Authors:** Cristian Abad-Coronel, Elena Carrera, Nancy Mena Córdova, Jorge I. Fajardo, Paulina Aliaga

**Affiliations:** 1 Prosthodontics Department, Faculty of Dentistry, Universidad San Francisco de Quito, Quito 170901, Ecuador; ecarrera@estud.usfq.edu.ec (E.C.); nmena@usfq.edu.ec (N.M.C.); 2 Department of Digital Dentistry and CAD/CAM Materials, Faculty of Dentistry, Universidad de Cuenca, Cuenca 010107, Ecuador; 3 Mechanical Engineering Faculty, Universidad Politécnica Salesiana, Cuenca 170517, Ecuador; jfajardo@ups.edu.ec; 4 Faculty of Dentistry, Universidad San Francisco de Quito, Quito 170901, Ecuador; paliaga@usfq.edu.ec

**Keywords:** CAD/CAM, CAD/CAM materials, rapid prototyping, interim restorations, fracture resistance

## Abstract

The aim of this study was to evaluate and compare the resistance to fracture of interim restorations obtained through additive techniques (3D impressions) and subtractive techniques (milling) using a computer-aided design and manufacture (CAD/CAM) system of a three-unit fixed dental prosthesis (FDP) to ascertain its clinical importance. (1) Materials and methods: In total, 40 samples were manufactured and divided into two groups (*n* = 20) using: (1) light-curing micro hybrid resin for temporary crowns and bridges (PriZma 3D Bio Prov, MarketechLabs, São Paulo, Brazil) for the rapid prototyping group (RP) and (2) a polymethylmethacrylate (PMMA) CAD/CAM disc (Vipiblock Trilux, VIPI, São Paulo, Brazil) for the computer-assisted milling (CC). The resistance to fracture was determined with a universal testing machine. (2) Results: The strength and the standard deviation for the computer-assisted milling group were higher (1663.57 ± 130.25 N) than the rapid prototyping (RP) group, which had lower values of (1437.74 ± 73.41 N). (3) Conclusions: The provisional restorations from the computer-assisted milling group showed a greater resistance to fracture than the provisional restorations obtained from the rapid prototyping group.

## 1. Introduction

According to *The Glossary of Prosthetic Terms* (GPT), an interim restoration is a “fixed or removable dental prosthesis that is designed in order to improve aesthetics, stabilization and/or function for a specified period of time, after which it must be replaced by a permanent dental prosthesis” [1]. The placement of interim restorations, which are considered intermediate treatments, becomes critical in cases of complete restorations, where several teeth are involved [2,3]. In the cases mentioned, these provisional restorations can be used for relatively long periods of time, from 6 to 12 weeks or even longer, ensuring the maintenance of the health of the remaining tissues, monitoring their stability, and making the necessary adjustments possible [4,5].

The use of interim restorations aims to: protect pulp and periodontal tissue, promote guided tissue healing, properly manage emergency profiles, and evaluate hygiene procedures, among others. With this type of restoration, in addition, an adequate occlusal scheme with the determined maxillomandibular relationships can be evaluated [2,4]. Regarding the material selection criteria for provisional restorations, the physical, mechanical, and handling properties must be considered, ensuring that the chosen material meets the specific requirements for each clinical case [3,4,5,6]. Another important factor to consider is the biocompatibility of a material with soft tissues as well as its bio tolerance because certain materials generate exothermic reactions that could be harmful [5].

It is also important to recognize the different requirements of interim restorations, such as: (1) the stabilization of teeth position, which requires marginal precision in addition to sufficient structural and wear resistance and (2) compliance with aesthetic characteristics while preserving their polish and shine [3,4,5]. Provisionalization is an important step to predict the final restorations, while the health of the abutments is restored and healing occurs in the areas of the pontics and periodontal tissues [2,6].

There are three big groups or techniques for making provisional restorations: (1) direct provisionalization in the mouth, on the abutments, or on the prepared teeth; (2) provisionals made indirectly; and (3) a mixed technique of indirect-direct provisionalization. Among the indirect techniques is the use of a CAD/CAM (computer-aided design–computer-aided manufacture) system, where certain processing errors (dosage, mixing, and material states) that can be part of a direct technique can be reduced. Through a CAD/CAM workflow system, a high-quality interim prosthesis is obtained, which can afford the demands of the patient and the clinician [7,8].

During the last decade, the use of CAD/CAM systems in dentistry has increased exponentially thanks to the advance in the acquisition of intraoral images, the development of design and manufacture technologies, and the presence of novel materials for dental restorations. With the help of these systems, restorations can even be performed in a single visit, improving the efficiency and quality of treatments [9,10].

In a digital workflow, obtaining the final product through the CAM process can be subtractive or additive. Within the subtractive process are milling and grinding, methods in which restorations are obtained from a monolithic block or disk of a certain material. Alternatively, the additive process is a manufacturing method where the final product is obtained by consecutively accumulating layers of material; for example, in the 3D printing method, a concentrated light beam is focused on the surface of a platform, and as the light beam attracts the object, the material polymerizes [9,10] in a technique known as rapid prototyping (RP). Currently, 3D printing has evolved with a wide variety of polymeric materials to obtain the final product [11].

Among the wide variety of selection materials for the creation of interim restorations in a conventional way are: (1) polymethylmethacrylate resins (PMMA), (2) polyethylmethacrylate resins (PEMA), (3) polyvinyl methacrylate resins, (4) bis-acrylic resins, and (5) urethane dimetacrylates. Each material used in the CAM has different processing parameters, so the system must be specifically adjusted. It is well established that the success of a prosthetic treatment performed using digital technology depends largely on the selection of the material. For interim restorations, polymer-based materials, such as PMMA, are among the CAD/CAM materials of primary choice. The PMMA blocks for the CAD/CAM systems have cross-linked structures, which provide greater advantages over conventional polymers [11,12]. Understanding the mechanical properties of a material is necessary to evaluate its behavioral conditions in clinical practice. Therefore, evaluating various mechanical properties such as flexural strength, hardness, impact resistance, and color stability becomes relevant [12,13,14,15].

Knowing the resistance to fracture and the microhardness of the materials for interim restorations is important, particularly when the patient must use the temporary restoration for a prolonged period, exhibits parafunctional habits, or when larger extension prostheses, such as fixed bridges, are planned [12]. According to Alp et al., the PMMA CAD/CAM exhibits a greater resistance to bending than bis-acrylic resin and conventional PMMA. Given this, the PMMA CAD/CAM has been positioned as a provisional prosthesis material for long-term use [11,12,14,16,17].

There is an abrupt increase in the use of design and manufacture of interim restorations using the CAD/CAM system due to its superiority to direct conventional techniques [18,19,20]. However, studies obtained on the comparison of the resistance to fracture of provisional restorations between subtractive and additive techniques are very limited [11,21,22]. That is why this study aimed to compare the resistance to fracture of provisional restorations obtained using additive techniques (3D impressions) and subtractive techniques (milling) with the CAD/CAM systems. The null hypothesis raised was that there would be no significant differences in the resistance to fracture between the group of provisional restorations obtained by milling and those obtained by 3D printing.

## 2. Materials and Methods

### 2.1. Sample Preparation

The materials used in the study are shown in Table 1.

**Figure 1:**
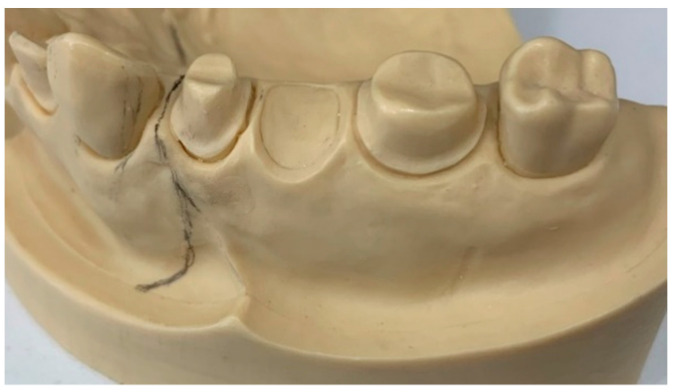
Maxillary typodont with preparations for a three-unit FDP.

A maxillary typodont with preparations for a three-unit fixed dental prosthesis (FDP) was used. The typodont with abutments at 16 and 14, and pontic at 15, had the following protocol: 2 mm occlusal reduction, 1.5 mm axial reduction, termination line with light chamfer, and parallelism between 6-degree axial walls and rounded edges. (Figure 1).

Two groups (twenty samples per each one) were obtained: one group of computer-assisted carving (CC) and another group of 3D printing of rapid prototyping (RP).

### 2.2. Sample Scanning and Design Process

**Figure 2:**
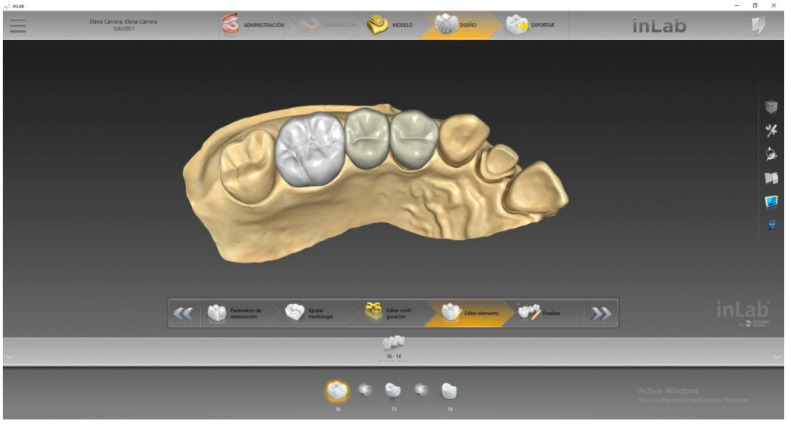
Design of a three-unit fixed dental prosthesis (FDP) using biogeneric modality.

Using a scanner (PrimeScan 2.0, Dentsply-Sirona, New York, NY, USA), a digital impression of the ready-made precast model was obtained. The model was digitized with a design software (InLAB 20.0, Dentsply-Sirona, New York, NY, USA). An indirect restoration of three units using a biogeneric modality was designed. (Figure 2).

### 2.3. Samples Materialization 

#### 2.3.1. Milling Process

**Figure 3:**
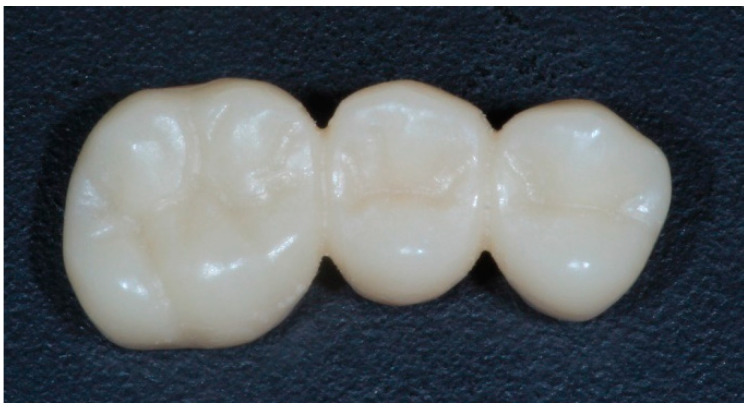
Sample of a milled FDP.

The design was transferred through CAM software (InLab CAM, 20, Dentsply-Sirona, New York, NY, USA) to the integrated milling unit (MCX5, Dentsply-Sirona, New York, NY, USA) to obtain the samples (*n* = 20) (Figure 3).

#### 2.3.2. Three-dimensional Printing Process

**Figure 4:**
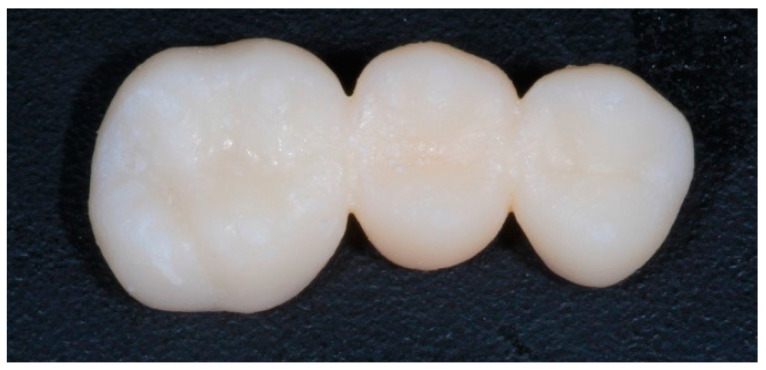
Sample of a 3D printed FDP.

Rapid prototyping samples (three-dimensional printing) were obtained by transferring the same CAD design to the CAM software of the 3D printer (MoonRay S, SprintRay, Los Angeles, CA, USA) as a file in STL (Standard Triangle Language) format (Figure 4).

#### 2.3.3. Postproduction

The printed samples went through a post-production process, where they were dipped and brushed with 90% isopropyl alcohol to remove resin residues. Subsequently, they underwent a photopolymerization process under UV (ultraviolet) light for 30 min, and, in addition, glycerin was applied to the entire restoration to ensure that the material reached complete polymerization.

### 2.4. Thermocycling

All samples were subjected to a thermocycling process; the cycles used were 5000 cycles. Thermal cycling with extreme temperatures of 5 °C and 55 °C in distilled water (residence time: 25 s, pause time: 10 s) was performed in the computerized thermocycling unit (Thermocycler™, SD Mechatronik, Feldkirchen-Westerham, Germany). The samples were placed in a thermal cycler container, and then dried and inspected for cracks, chips, or fractures after each loading phase.

### 2.5. Fracture Strength Test

A master typodont was printed with model resin (Model-Gray, SprintRay, Los Angeles, CA, USA) with a resolution of 50 microns for subsequent testing by means of a 3D print of the STL file obtained from the initial scan of the prepared original typodont.

The fracture strength of the provisional (FDP) was tested using a semi-clinical experimental design under ambient laboratory conditions. The 3D printed resin master typodont was fixed on the platform of the universal testing machine (Shimadzu AGS-X series Universal Testing Machine; Shimadzu, Tokyo, Japan). No fixing agent was used to bond the FDP to the abutments of the master resin typodont.

**Figure 5:**
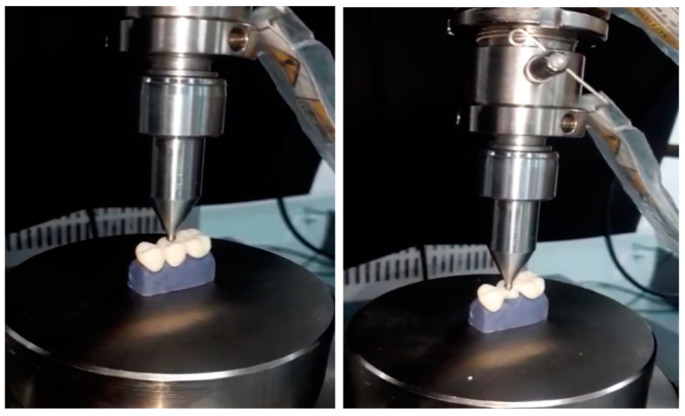
Fracture strength test in a universal testing machine.

Specimen dimensions were visually inspected following a uniform design. The fits of all specimens were verified with an explorer and a fit tester. After this, the fracture test was carried out by means of the load compression mode applied occlusally on the pontic surface using a 3 mm diameter metal sphere at a speed of 0.5 mm/min until failure occurred. The maximum of the fracture strength was recorded in Newtons (N) (Figure 5).

### 2.6. Evaluation of Fracture Mode

The fracture surfaces of the samples after loading were observed in a stereo microscope (OlympusSZX7, Olympus, New York, NY, USA).

### 2.7. Statistical and Analysis Evaluation

A file was prepared with the descriptive values of the strength and displacement variables for each group using measures of central tendency (mean) and measures of dispersion (standard deviation, coefficient of variation, minimum, and maximum). Prior to contrasting the hypotheses, a normality test was carried out to know the distribution of the data and determine the appropriate statistical test. The level of significance used in all tests was 5%. The statistical analysis was carried out using a statistical program (SPSS v.25, IBM, New York, NY, USA).

## 3. Results

### 3.1. Descriptive Analysis

**Figure 6:**
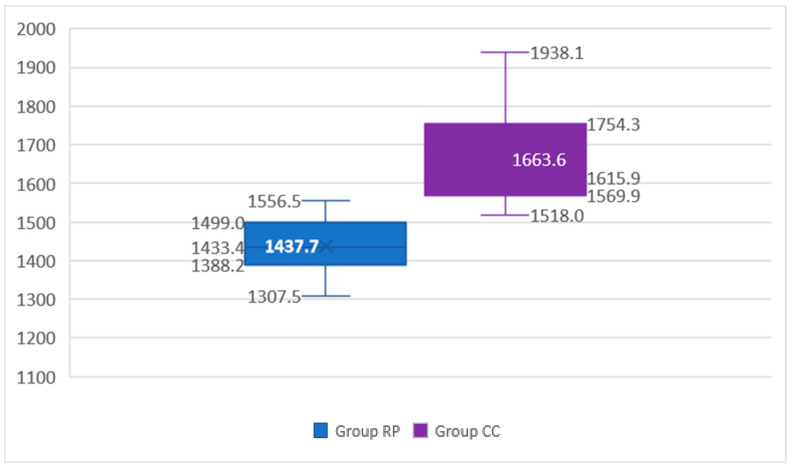
Box plot for the fracture strength variable for each group.

Table 2 shows a descriptive analysis of the variables to study the resistance to fracture of an interim restoration obtained by milling and another by 3D printing. The mean strength and standard deviation for the RP group is lower (1437.74 ± 73.41 N) than the mean strength and standard deviation of the CC group (1663.57 ± 130.25 N). It can also be observed that the maximum value of the strength obtained by 3D printing is very close to the minimum value from the restoration obtained by the milling technique. The coefficients of variation for 3D printing and milling are low, 5.11% and 7.83%, respectively, which indicates low dispersion in the different replicates with respect to the mean value (precision) (Figure 6).

### 3.2. Inferential Analysis

The results of the hypothesis test considering the strength variable were significant (*p* Value < 0.05), representing that the null hypothesis was rejected and concluding that there are differences between the groups studied. The strength applied to the CC group is higher.

**Figure 7:**
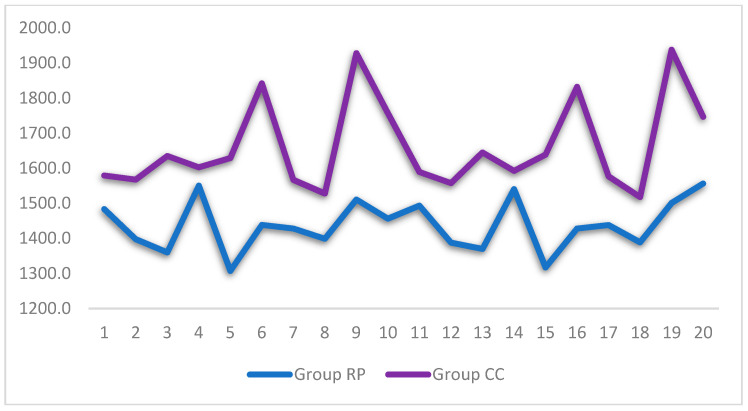
Line graph for the strength variable, according to the group. Note: the lines show the distribution of the different replicas in each group.

Figure 7 shows the differences in the measurements obtained by each technique (milling and 3D printing). It was observed that the CC group obtained higher values than the 3D technique in terms of strength.

**Figure 8:**
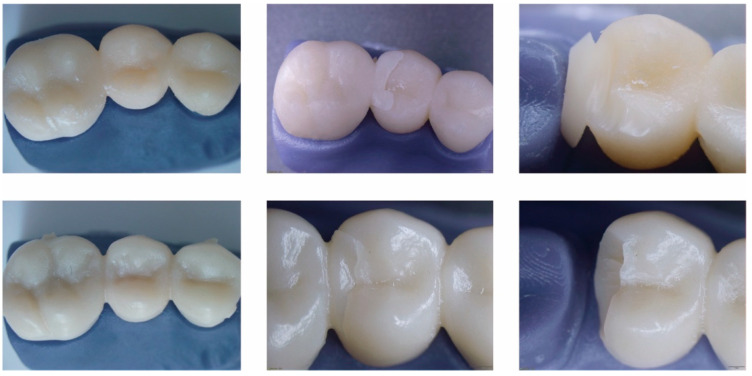
Image of fracture of bridge in pontic zone. Upper images correspond to printed restorations and lower images to milled restorations.

Figure 8 shows the images obtained from the passage of the samples through a stereomicroscope after the fracture resistance test. The upper images correspond to the group of printed restorations, while the lower images correspond to the group of milled restorations.

## 4. Discussion

There is limited information comparing the fracture strength of 3D printed interim restorations with those obtained using subtractive CAD/CAM techniques (Table 3). Some studies have been based on examining the internal fit of interim restorations. The present study was carried out with the aim of evaluating the resistance to fracture of interim restorations obtained using subtractive and additive techniques. The study proposed a null hypothesis where there would be no significant difference between the fracture strength of 3D printed and milled interim restorations.

Temporary restorations have become one of the essential components of rehabilitative treatment with fixed prosthodontics, which has to satisfy biological, mechanical, and aesthetic requirements. Among the mechanical requirements is the resistance to functional loads and traction forces, among others [2,3,4,5].

CAD/CAM technology is one of the fundamental pillars within current dentistry for the manufacture of interim restorations, demonstrating great clinical success attributed to the technological advances and the series of innovative materials currently being used [6,7]. To solve certain drawbacks of this technology, such as material waste, 3D printing technology has recently interfered with the manufacture of interim restorations, as an alternative to the subtractive technique, because of its ability to manufacture unlimited objects, due to manufacturing time, called rapid prototyping [7,8,9].

In the printing process, a concentrated light beam is focused on the surface of a platform. As the light beam attracts an object, the material polymerizes layer by layer until a shape is produced in three dimensions. This requires less material and reduces the costs that its manufacture could entail [20,21,22]. Several authors have mentioned that the three-dimensional printing technique improves the mechanical properties of a final restoration due to the fact that it does not present cracks: a high level of construction is generated in terms of resolution, and a high resistance is attributed to the chemical bond between layers. This is in contrast to a restoration obtained using subtractive techniques, where the resistance to fracture is directly affected by the machining of the blocks required to achieve milled restorations [20,21,22,23,24].

In the case of this study, the null hypothesis was rejected because a significant difference was evidenced between the resistance to fracture of both the milled and printed interim restorations, which showed that the restorations obtained by milling had a greater resistance to damage fracture than the printed prostheses. In the microscopic detail of the fractures, the fracture is much sharper in the milled restorations, while in the printed ones, the line is more irregular with areas of tearing. This shows better resilience of milled crowns to applied load and catastrophic failure. The printed crowns show more resilience to the applied load.

When evaluating the obtained results for the resistance to fracture, the average values of the resistance to fracture for the group of milled restorations were 1663.57 ± 130.25 N, while the average value registered for the group of impressed restorations was 1437.74 ± 73.41 N. The milled restorations group presented a statistically significant difference compared to the impression restorations group (*p* ≤ 0.05).

The values obtained by this study differ from the findings of Ibrahim [19,20,23,25,26,27], who showed that the group of printed provisional restorations registered a mean value of the resistance to fracture much higher than the group of milled provisional restorations, showing values up to 1226.48 N. It should be noted that this study was carried out not with bridges, but with a unitary crown of piece 26, which can affect the results by having connector areas and a pontic [19,20,23,28,29,30,31]. The author established that the higher fracture resistance in the group of printed restorations could be attributed to the nature by which the 3D structures were created. The higher values of the resistance to fracture in the group of printed restorations could also be due to the vertical orientation used in the study [19,20,26,27,32,33]. Furthermore, it is also suggested that the higher resistance to fracture of the group of impressed restorations could be attributed to the rather thin thickness of the impression layer used in the study. This finding on layer thickness agrees with Tahayeri [34,35,36,37], where it was shown that the thickness of the resin layer can contribute directly to the final mechanical properties of restorations and found that the lower the layer thickness, the more interfaces are generated, and a better degree of polymerization directly affects mechanical performance [37]. Taking this factor into account, the specimens in our study were printed at a thickness of 50 microns. Another factor that, according to Tahayeri [37], influences the quality of restorations, is the post-production process, where it is suggested that printed restorations have a higher degree of conversion and a decrease in residual monomers, thereby improving the toughness and resistance to fracture of the material [37]. However, the industrial manufacturing processes of monolithic discs ensure high degrees of monomer conversion and better reticular compaction of the PMMA structure, which allows a greater resistance [37].

Conversely, the results obtained by this study are similar to the findings of Hazeveld [38], where it was found that the printed restorations presented a lower resistance to fracture than the milled interim restorations [38]. The authors suggested and attributed this result to the shrinkage that the material undergoes during construction and post-production. In turn, they attributed this to the manipulation of data in the STL file, where conversion and formatting were generated and could result in changes [38,39,40,41].

Likewise, the results of the present study are akin to those of Digholkar [10], a study where flexural strength values were analyzed and compared in provisional crowns manufactured through three different techniques. The group of milled provisional restorations presented a higher value (104.20 MPa) than the value obtained from the group of restorations using conventional manufacturing techniques (PMMA) (95.58 MPa) and registered the lowest values of flexural strength for the group of restorations obtained by 3D impression (79.54 MPa) [10,42,43,44,45].

Moreover, in the study carried out by Merve [46], where they studied two different groups of materials for use in interim restorations (milled PMMA and polylactic acid for printed restorations), the fracture resistance values obtained from the group of the milled PMMA interim restorations were located at 752.00 N, while the value for the impression interim restorations was 681.00 N. These values give results of a greater resistance to fracture for the interim restorations obtained using the milled technique, similar to the results obtained by the present study. The values presented by Merve, however, are lower; this may be due to several factors, including that the work carried out by the author, Merve, was on individual crowns, while the present study was carried out on a three-unit bridge [46]. A comparative table of results of several studies and our results is showed in table.

The present study reveals that the interim restorations using the milling technique have a greater resistance than the provisional restorations made using 3D printing. When a comparison was made between temporaries made with CAD/CAM technology and conventional techniques, one study, which analyzed the fracture resistance of conventionally used materials, presented values that were between 40 and 50 N, really low compared to those obtained by both CAD/CAM techniques in our study [47]. Increasing the fracture resistance of temporary restorations using innovative CAD/CAM techniques in the workflow has the potential to generate greater productivity for the clinician. The low values obtained from the provisional restorations made by 3D printing show that they can be used, as the author Merve cites, in cases in which they will not be subjected to high chewing loads, providing a second quality clinical option [46,47].

This study had several limitations. The conditions under which the experimental part was carried out were in vitro and a simulation to the oral environment was not generated. The resistance to fracture test was carried out using the three-point test, which could differ from a cyclical load that would have simulated the masticatory load in a better way. It is also important to mention that the material for each manufacturing category was different, and it is necessary to analyze more thoroughly and chemically the components of each of the materials that could cause them to differ in their final results. More materials could be included to establish differences between the different manufacture techniques.

There is a definite shortage of literature on the subject, so more studies are required to analyze the mechanical properties of both milled and printed interim restorations. In turn, it is important that future work also investigate the resistance to wear, fatigue, hardness, micro-, and nanohardness, which together would include a better characterization of the material. Finally, analyzing chromatic properties and their color stability would also be a contribution to establish differences between milled and printed temporary materials.

## 5. Conclusions

1. The interim restorations materialized by a milling technique showed a higher resistance to fracture compared to the provisional restorations obtained by 3D printing.
2. The fabrication of interim restorations using a subtractive technique, or milling technique, could be considered a reliable and conservative method for the production of stronger provisional restorations.
3. Although the strength values of the printed restorations were lower, the rapid prototyping material could be considered in certain scenarios with reduced chewing loads and surfaces exposed to less stress.

## Figures and Tables

**Table 1:** Materials used in this study.

Product Name	Brand/Manufacturer	Batch	Base Material
Vipiblock Trilux	VIPI	0000054908	PMMA CAD/CAM Disk
PriZma 3D Bio Prov	MarkertechLabs	E1488	Light-Curing Micro Hybrid Resin

**Table 2:** Descriptive summary of the strength variable for each group.

Group	Statistics	Strength (N)
RP (rapid prototyping)	Mean	1437.74
	Standard deviation	73.41
	VC	5.11%
	Minimum	1307.49
	Maximum	1556.45
CC (computer-assisted milling)	Mean	1663.57
	Standard deviation	130.25
	VC	7.83%
	Minimum	1517.98
	Maximum	1938.09

Note: Variation coefficient (VC).

**Table 3:** Summary articles related to the study topic.

Author	Objective	Resistance to Fracture/Milled Restorations	Resistance to Fracture/Printed Restorations
Ibrahim, et al. (2020)	Resistance to fracture between milled and printed restorations	933 N	1226.48 N
Suralik, et al. (2020)	Investigate the impact of the manufacturing technique on the resistance to fracture of the 3-unit temporary resin fixed dental prostheses	412.03 N	603.33 N
Digholkar, et al. (2016)	Flexural strength values in temporary crowns manufactured through 3 different techniques	104.20 MPa	79.54 MPa
Merve, et al. (2021)	Measurement of fracture strength and fracture modes	752.00 N	681.00 N

## Data Availability

https://onedrive.live.com/?cid=ae30f444ffec7d8a&id=AE30F444FFEC7D8A%211607&authkey=!ADCxLwEsFSgHC5c (accessed date: 12 December 2021).
